# Municipal Supervision of Restaurants

**Published:** 1905-03

**Authors:** 


### MUNICIPAL SUPERVISION OF RESTAURANTS.

The city corporation of London has taken upon itself the supervision of the sanitary arrangements, storing, and cooking of food in restaurants. Certificates are granted by the Health Officer to proprietors who conduct their places properly, and nearly 1,000 London restaurants have been inspected. The proprietors instead of resenting the inspection have welcomed the inquiry, and have been eager to secure the certificate of the corporation, which invites trade, as it is accepted as a guarantee that the food is wholesome and prepared with cleanliness. It seems too bad that our government is not paternal enough to adopt so excellent a system, which insures the use of good food and cleanliness in its preparation.—*Medical Age.*

